# Assessing pre- and postoperative activity levels with an accelerometer: a proof of concept study

**DOI:** 10.1186/s12893-017-0223-0

**Published:** 2017-05-12

**Authors:** Eva van der Meij, Hidde P. van der Ploeg, Baukje van den Heuvel, Boudewijn J. Dwars, W. J. H. Jeroen Meijerink, H. Jaap Bonjer, Judith A. F. Huirne, Johannes R. Anema

**Affiliations:** 1 Department of Public and Occupational Health, EMGO+ Institute for Health and Care Research, VU University Medical Center, van der Boechorsstraat 7, 1081 BT Amsterdam, The Netherlands; 2 Department of Obstetrics and Gynaecology, VU University Medical Center, Amsterdam, The Netherlands; 3 Department of Surgery, Jeroen Bosch ziekenhuis, Den Bosch, The Netherlands; 4 Department of Surgery, Slotervaartziekenhuis, Amsterdam, The Netherlands; 5 Department of Surgery, VU University Medical Center, Amsterdam, The Netherlands

**Keywords:** Accelerometer, Physical activities, Postoperative recovery, Abdominal surgery, Inguinal hernia surgery, Cholecystectomy, Hysterectomy, Adnexal surgery

## Abstract

**Background:**

Postoperative recovery after abdominal surgery is measured mostly based on subjective or self-reported data. In this article we aim to evaluate whether recovery of daily physical activity levels can be measured postoperatively with the use of an accelerometer.

**Methods:**

In this multicenter, observational pilot study, 30 patients undergoing laparoscopic abdominal surgery (hysterectomy, adnexal surgery, cholecystectomy and hernia inguinal surgery) were included. Patients were instructed to wear an Actigraph wGT3X-BT accelerometer during one week before surgery (baseline) and during the first, third and fifth week after surgery. Wear time, steps taken and physical activity intensity levels (sedentary, light, moderate and vigorous) were measured. Patients were blinded for the accelerometer outcomes. Additionally, an activity diary comprising patients’ self-reported time of being recovered and a list of 18 activities, in which the dates of resumption of these 18 activities were recorded after surgery, was completed by the patient.

**Results:**

Five patients were excluded from analyses because of technical problems with the accelerometer (*n* = 1) and protocol non-adherence (*n* = 4). Light, moderate, vigorous, combined moderate and vigorous intensity physical activity (MVPA), and step counts showed a clear recovery curve after surgery. Patients who underwent minor surgery reached their baseline step count and MVPA three weeks after surgery. Patients who underwent intermediate surgery had not yet reached their baseline step count during the last measuring week (five weeks after surgery). The results of the activity diaries showed a fair agreement with the accelerometer results (Cohens Kappa range: 0.273-0.391). Wearing the accelerometer was well tolerated and not regarded as being burdensome by the patients.

**Conclusions:**

The accelerometer appeared to be a feasible way to measure recovery of postoperative physical activity levels in this study and was well tolerated by the patients. The agreement with self-reported physical recovery times was fair.

**Electronic supplementary material:**

The online version of this article (doi:10.1186/s12893-017-0223-0) contains supplementary material, which is available to authorized users.

## Background

Postoperative recovery is an essential component of surgical therapy. Innovation of surgical techniques and the development of interventions aiming to enhance the recovery process, necessitate tools to measure recovery [1–5]. A frequently used standard to measure postoperative recovery is length of hospital stay, because it provides us with a well-defined and objective definition [6]. However, an increasing number of operations are performed as day surgeries and hospital stay has decreased generally over the last decade [7]. Patients are not fully recovered at time of discharge and subsequently, hospital stay does not correlate with postoperative recovery. Currently, a wide variation of instruments and outcomes are used to determine postoperative recovery, such as quality of life, satisfaction, pain, recovery indices and return to normal activities [1, 8–10]. Unfortunately, all of these measuring instruments are subjective and self-reported and therefore prone to measurement bias [1].

In physical activity research, accelerometers are used as an objective measure of physical activity and sedentary behaviors [11, 12]. Furthermore, activity monitors such as pedometers and the now widely commercially available wearables are used to promote physical activity, for example as behavior change tool in trying to prevent and manage obesity [13, 14]. Promoting physical activity is an important element in medical care as well, for example in the prevention of postoperative complications such as pneumonia, decubitus and deep venous thrombosis [15–17]. The use of accelerometers in postoperative care seems therefore logical, not only to measure postoperative physical activity levels, but also as a tool to stimulate physical activities after surgery.

Some earlier research has been carried out with accelerometers in perioperative care, but these studies have focused on a relatively short postoperative period or used the accelerometer as an intervention to stimulate physical activities [18–20]. Therefore, data representing the normal recovery process measured with an accelerometer are lacking. In this observational pilot study we will measure physical activities during four moments of one week each in the postoperative period. We aim to evaluate:

1. Whether recovery of physical activity levels can be measured postoperatively with the use of an accelerometer.
2. Whether the physical activity results measured with the accelerometer correspond with self-reported recovery of physical activity levels.
3. Whether the use of an accelerometer in the postoperative course is feasible and accepted by patients.

## Methods

### Study design

A multicenter, observational pilot study was conducted including 30 patients. Two teaching hospitals in Amsterdam, the Netherlands participated in the study. All patients waiting for surgery, who met the inclusion criteria, were approached for participation and signed informed consent. Patients received a gift card of 50 euros after completing the study. The study was conducted in accordance with the STROBE statement [21]. The study was approved by the local medical ethics committee of the VU medical center with registration number 2014.364.

### Population

Patients between 18 and 75 years old, who were on the waiting list for the following type of surgeries were included: laparoscopic hysterectomy, laparoscopic adnexal surgery, laparoscopic cholecystectomy and laparoscopic inguinal hernia repair. These procedures were selected since we aimed to include a various sample of procedures because of the proof of concept design of the study and these are commonly applied surgical and gynecological procedures in the Netherlands. Laparoscopic hysterectomy was defined as intermediate surgery and laparoscopic adnexal surgery, laparoscopic cholecystectomy and laparoscopic inguinal hernia repair as minor surgery. This subdivision is based on a classification which has been used previously in gynecologic surgery [22, 23]. The general surgical procedures were classified in line with these classifications. We based the latter on Delphi based recommendations among experts for resumption of normal activities after various surgical procedures in gynecology and general surgery [24, 25]. Exclusion criteria were: (suspicion of) malignancy, deep infiltrating endometriosis, less than one week of waiting between identification and surgery, lack of understanding of the study information or insufficient Dutch language proficiency.

### Outcome measures

#### Physical activities – Accelerometer

We used the Actigraph wGT3X-BT accelerometer to measure physical activity (Actigraph, Pensacola, FL, USA), which is a small lightweight device worn on the hip with an elastic belt. Patients were asked to wear the accelerometer for seven consecutive days during the week before surgery (T0), one week after surgery (T1), three weeks after surgery (T2) and five weeks after surgery (T3), resulting in four measuring moments of one week each. Patients were asked to wear the accelerometer during waking hours on the right hip, except during water based activities. The Actigraph does not provide feedback to the patient. The sensor of the accelerometer provides raw acceleration data and also converts accelerations into activity counts based on a company algorithm. Activity counts are summed up and stored over a set time period (epoch). Accelerometer data was cleaned in 60 second epochs in order to capture health enhancing physical activities in line with the protocol used in NHANES [26]. Non-wear time was defined as ≥ 60 minutes of no activity (consecutive zeroes, allowing for two interruptions of <100 counts). A valid day was defined as having a minimum of 10 h per day wear time, with the exception for the first week after surgery when a valid day was defined as six hours of wear time per day. This was done as patients were more bedridden in this week and often did not wear the accelerometer when in bed. Participants needed a minimum of four valid days per measuring week, to be included in the analyses. Steps taken and time spent in different intensity levels were used as outcomes. Existing cut-off points were used for the vertical axis of the accelerometer, in order to determine time spent in sedentary time (<100 counts/min), and light- (100–2019 counts/min), moderate- (2020–5998 counts/min) and vigorous intensity physical activity (≥5999 counts/min) [26].

#### Physical activities – Activity diary

Patients completed an activity diary after surgery, in which they recorded the first date they were able to perform a specific activity. This comprises a newly developed list of 18 activities (Additional file 1). Patients were instructed to fill in the dates on which they felt they had returned to their normal activity level for each of the 18 activities on the list. Patients were provided with the option to fill in ‘not applicable’ where appropriate. In addition the list contained two questions ‘when did you feel physically recovered’ and ‘when did you feel fully recovered (physically and mentally)’. After five weeks patients were asked to return the list. When patients had not returned to their normal activity level regarding an activity, the date was censored on five weeks after surgery. The list was based on an earlier study of our research group in which patients could make their own recovery advice based on recommendations which were developed in a Delphi study [24, 25].

#### Feasibility - Feasibility questionnaire

We evaluated the feasibility of the accelerometer with a number of questions five weeks after surgery (Additional file 2). These questions concerned compliance, burden and usability of the accelerometer.

### Statistical analyses

Mean step count and physical activity intensity level were calculated per measuring day and per measuring week, using only valid days. The definition for recovery of physical activity levels was that the mean activity level was at 90% or above of the mean baseline activity level, a definition which is commonly used in similar type of research [19]. This was calculated for both step count and mean moderate physical activity intensity (MVPA). Comparing normally distributed scale variables between two subgroups was performed using the independent-Samples *T* test. When a scale variable was not normally distributed, the Mann-Whitney *U* test was used. Comparing nominal variables between two subgroups was done using the *X* ^2^ test. Repeated-measures ANOVA were used to compare the mean counts of the accelerometers between the different time points. A significance level of *p* < 0.05 was considered. Agreement between the accelerometer and the activity diary was quantified using Cohens kappa. A Cohen kappa of ≤ 0 was considered as no agreement, 0.01–0.20 as none to slight agreement, 0.21–0.40 as fair agreement, 0.41– 0.60 as moderate agreement, 0.61–0.80 as substantial agreement, and 0.81–1.00 as perfect agreement [27].

## Results

### Participants

**Fig. 1:**
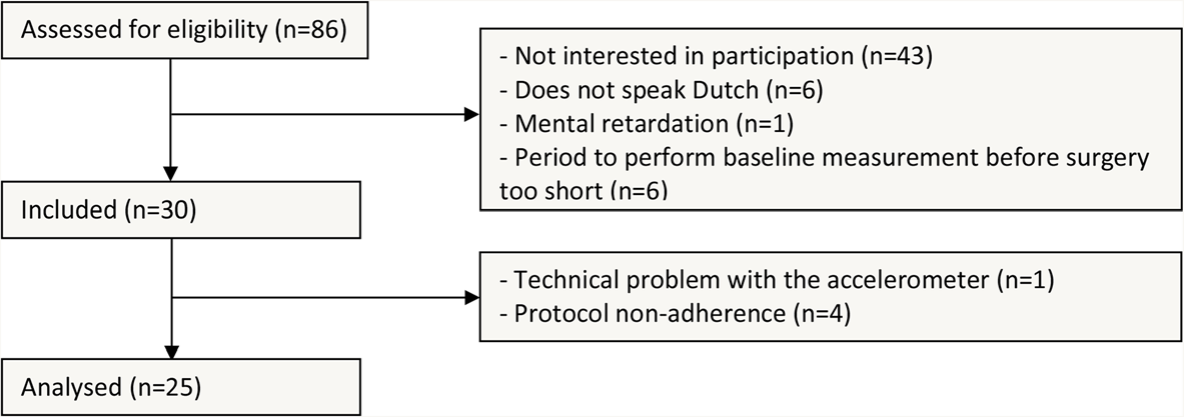
Flowchart

Between September 2014 and July 2015 we identified 106 patients from the surgery waiting list. In 20 patients surgery had already been carried out (*n* = 11), surgery had been cancelled (*n* = 7), or we had not been able to reach them in time (*n* = 2). Of the 86 patients assessed for eligibility, 30 patients (34.9%) participated in the study. The main reason for non-participation was that patients were not interested in study participation (Fig. 1). Comparison between the patients who participated and those who did not, revealed no statistically significant differences regarding demographic characteristics (gender, age, social economic status) and health related characteristics (type of surgery, American Society of Anesthesiologists (ASA) classification and body mass index (BMI)).

All 30 included patients completed the study. Data of 25 patients were used for analyses (Table 1). Data of five patients were not used for analyses due to an accelerometer error (*n* = 1) or not meeting the accelerometer wearing criteria (*n* = 4). Most patients were female (76.7%), the mean age was 44.3 years old and most patients were employed (83.3%). Seventeen patients underwent minor surgery and 13 patients underwent intermediate surgery. There were no major differences in baseline characteristics between patients who were analyzed and those whose data were not used for the analyses, except for BMI which was higher in the group of patients excluded from analyses.

**Table 1:** Characteristics of the analyzed patients

	Analyzed patients (*n* = 25)	Patients excluded from analyses (*n* = 5)
Gender (n %)		
- Male	6 (23.3%)	1 (20.0%)
- Female	19 (76.7%)	4 (80.0%)
Age (mean sd)	45.16 (8.96)	42.6 (7.16)
SES (mean sd) [31]	0.82 (1.05)	−0.33 (0.78)
Level of education (n %)		
- Low	5 (20.0%)	1 (20.0%)
- Medium	10 (40.0%)	2 (40.0%)
- High	10 (40.0%)	2 (40.0%)
Employment status (n %)		
- Employed	21 (84%)	4 (80%)
- Unemployed	4 (16%)	1 (20%)
Type of surgery (all laparoscopic) (n %)		
- Minor	13 (52%)	4 (80%)
- Adnexal surgery	6	1
- Inguinal hernia repair	4	1
- Cholecystectomy	3	2
- Intermediate (hysterectomy)	12 (48%)	1 (20%)
ASA classification (mean sd)	*n = 20* 1.20 (0.41)	*n = 4* 2.25 (0.50)
BMI (mean sd)	25.31 (4.22)^a^	29.12 (5.40)
Sport (n %)^b^		
- Yes	18 (72%)	3 (60%)
- No	7 (28%)	2 (40%)

*SES* Social Economic Status. Scores are based on geographic location, *ASA* American Society of Anesthesiologists classification, *BMI* Body Mass Index

^a^Significantly lower in the analyzed patients compared to the patients whose data were not used for the analyses

^b^Patients were asked on baseline whether they perform any type of sport in daily life

### Results of the accelerometer

Table 2 presents the accelerometer results per measuring week for the 25 patients. The *p*-values indicate whether the values of each measuring week differ significantly from each other. Values highlighted with a * differ significantly in comparison to the value of T0 (baseline measurement). The mean number of valid days per week was similar for each measuring week. The mean wear time per day differed significantly between the different measuring weeks (*p* < 0.001) with the longest wear time during T0. Of the different physical activity intensity levels, light, moderate, vigorous, combined moderate and vigorous activities (MVPA) and step count differed significantly per measuring week, thus representing the clearest recovery curves after surgery. Mean min/day moderate physical activity and MVPA on T3 were not significantly different from T0, indicating that baseline levels were reached at this moment.

**Table 2:** Mean accelerometer data (*n* = 25), based on valid days

	One week before surgery (T0)	One week after surgery (T1)	Three weeks after surgery (T2)	Five weeks after surgery (T3)	*P*-value for time trend ‡
Mean min/day **wear time** per day (SD)	869 (64.18)	709 (109.71)*	815 (71.62)*	822 (71.72)*	<0.001
Mean number of **valid days** per week (SD) †	6.24 (1.10)	5.76 (1.16)	6.16 (1.03)	6.20 (0.96)	0.175
Mean min/day **sedentary physical activity** (SD)	548 (101.67)	554 (83.62)	549 (78.49)	534 (91.81)	0.674
Mean min/day **light physical activity** intensity (SD)	294 (85.86)	148 (76.98)*	248 (94.89)*	268 (85.70)*	<0.001
Mean min/day **moderate physical activity** intensity (SD)	26.08 (19.23)	6.53 (8.93)*	17.76 (15.16)*	19.47 (14.94)	<0.001
Mean min/day **vigorous physical activity** intensity (SD)	1.02 (2.30)	0.08 (0.28)	0.41 (1.19)	0.31 (1.31)	0.089
Mean min/day **moderate and vigorous physical activity intensity (MVPA)** (SD)	27.10 (19.46)	6.61* (9.08)	18.17* (15.53)	19.78 (14.94)	<0.001
Mean **step count** per day (SD)	7634 (3343.25)	2775^*^ (1815.71)	5912* (3134.72)	6464* (2781.38)	<0.001

* significant different compared to T0 (Bonferroni corrected *p*-value < 0.05)

† A valid day was defined as having a minimum of 10 h per day wear time, except for the first week after surgery (T1) when a valid day was defined as 6 h of wear time per day

‡ The *p*-values indicate whether the values of each measuring week differ significantly from each other

**Fig. 2:**
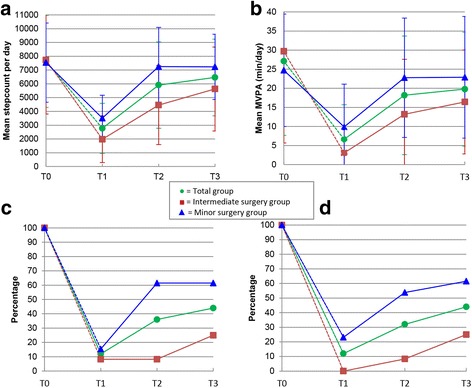
Results of the accelerometer. Figure legend: Intermediate surgery group: laparoscopic hysterectomy. Minor surgery group: adnexal surgery, cholecystectomy, inguinal hernia repair as minor surgery (all laparoscopic). **a**: Mean step count per measuring week. **b**: Mean MVPA per measuring week. **c**: Percentage of patients who reached baseline step count per measuring week. **d**: Percentage of patients who reached MVPA per measuring week. T0: one week before surgery, T1: one week after surgery, T2: three weeks after surgery, T3: five weeks after surgery

In Fig. 2 the mean step count (2A) and MVPA (2B) levels per measuring week are graphically displayed, for the total group and according to type of surgery. For the group of patients who underwent minor surgery, mean step count and MVPA differed significantly on T1 compared to T0, but this was no longer the case on T2 (*p* = 0.650 and *p* = 0.543 respectively), indicating that these group of patients had achieved their mean baseline step count and MVPA three weeks after surgery. However the group of patients who had undergone intermediate surgery had not even achieved their mean baseline step count and MVPA five weeks after surgery (difference between T3 and T0 *p* = 0.007 and *p* = 0.022 respectively). When determining whether or not each individual patient has achieved his or her own baseline step count (2C) or MVPA (2D), the graph shows a different pattern. In contrast to what graph 2A and 2B suggests, graphs 2C and 2D show that only 44% of the patients had reached their baseline step count and MVPA within five weeks after surgery (61.5% (8/13) of the patients who underwent minor surgery and 25% (3/12) for the intermediate surgery patients). This illustrated the individual variations between patients, with some patients surpassing their baseline levels (*n* = 8), which increases the mean group levels. Patients who did not reach their baseline step count five weeks after surgery (*n* = 11) on average achieved 69.45% of their baseline step count (range 43.57–89.40%). For MVPA this was 50.7% with a range of 17.95–89.43%.

**Fig. 3:**
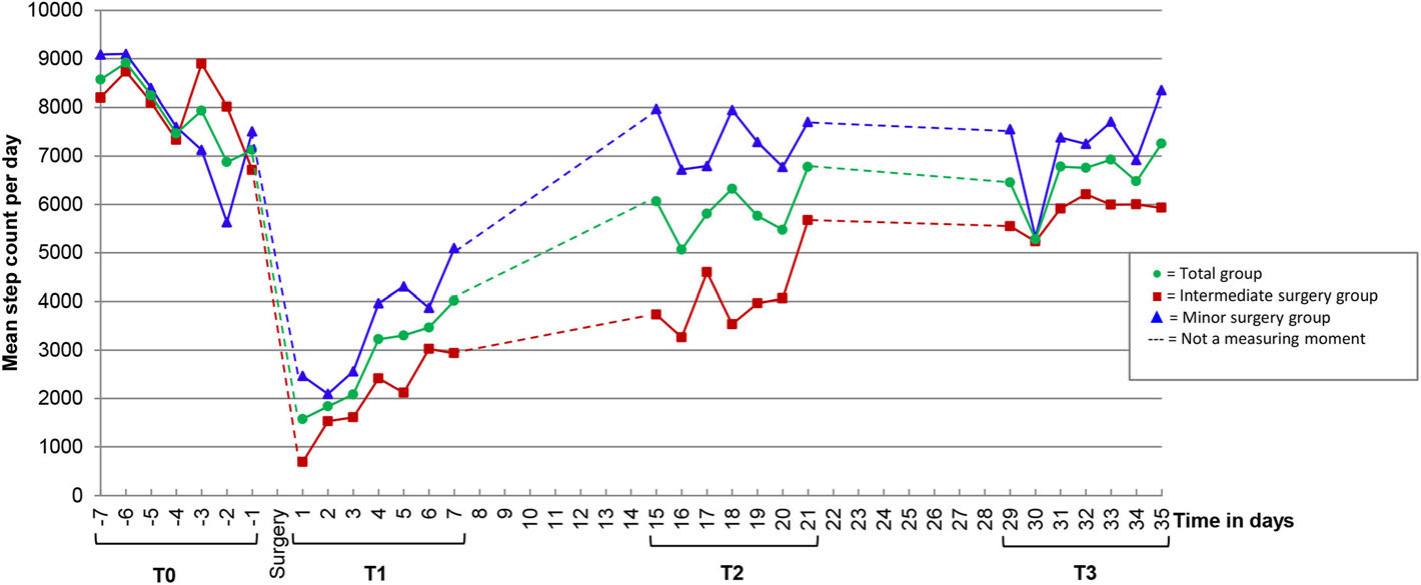
Mean step count per day according to type of surgery. Figure legend: Intermediate surgery group: laparoscopic hysterectomy. Minor surgery group: adnexal surgery, cholecystectomy, inguinal hernia repair as minor surgery (all laparoscopic). T0: one week before surgery, T1: one week after surgery, T2: three weeks after surgery, T3: five weeks after surgery

Figure 3 presents the daily variation in mean step count for each assessment week. This clearly shows the expected within week variation of activity levels but also reveals a clear recovery curve in the first week after surgery.

**Fig. 4:**
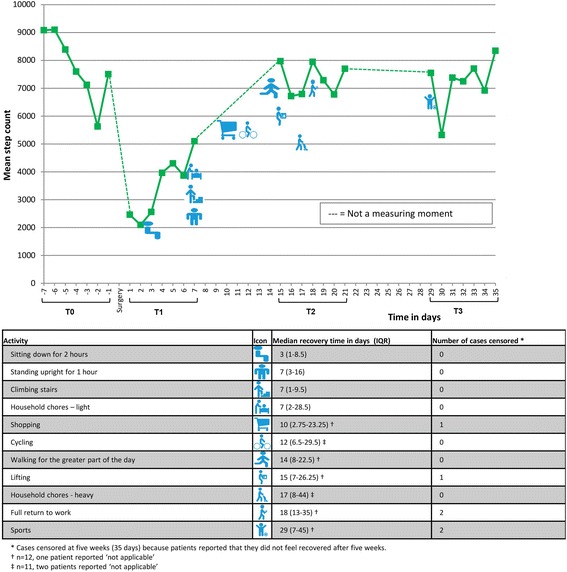
Mean step count per day and median reported recovery times (minor surgery group). Figure legend: T0: one week before surgery, T1: one week after surgery, T2: three weeks after surgery, T3: five weeks after surgery

Twenty-four patients returned their activity diary at the end of the study. Twenty five percent of the patients (6/24) resumed all the activities which were applicable to them five weeks after surgery. This showed a fair agreement with reaching baseline step count five weeks after surgery (Cohens kappa 0.391) and reaching MVPA five weeks after surgery (Cohens kappa 0.273). In addition patients were asked whether they felt physically recovered and fully mentally and physically recovered after five weeks. 45.8% (11/24) reported that they felt physically recovered after five weeks. This showed moderate agreement (Cohens kappa 0.497) with reaching baseline step count after five weeks and fair agreement with reaching MVPA after five weeks (Cohens kappa 0.239). 37.5% (9/24) reported that they felt fully mentally and physically recovered after five weeks. This matched fair with the accelerometer data. (Cohens kappa of 0.319 for reaching baseline step count and Cohens kappa of 0.391 for reaching MVPA after five weeks). A selection of the most relevant activities of the activity diary are presented in Fig. 4 (12 out of 20). The majority of the patients who underwent intermediate surgery did not resume the majority of the activities by the end of week 5. Therefore, the outcomes of the intermediate surgery group were not included in Fig. 4. In Fig. 4 the median recovery times for the selected activities of the patients who underwent minor surgery are plotted against the daily step count levels. Return to seven out of 12 activities had a median time between one and two weeks after surgery, the weeks in which the largest increase in step count was detected.

We evaluated the feasibility of the accelerometer with a number of questions among the participants at the end of the study. One participant did not respond. Of the remaining 24 participants, 23 (95.8%) reported that it had been clear to them how to use the accelerometer. None of the patients found it burdensome to wear the accelerometer. Ten patients (41.7%) indicated that they had failed to wear the accelerometer for one or two days. Reasons were that they had forgotten it (*n* = 6), because they had stayed in bed the whole day (*n* = 3) and that it had been too painful to wear the accelerometer at these moments (*n* = 1).

## Discussion

Results of this proof of concept study show that the accelerometer provides a feasible and objective method to measure recovery of physical activity levels after various forms of abdominal surgery. The group of patients who underwent minor surgery reached their mean baseline step count and MVPA three weeks after surgery. The group of patients who underwent intermediate surgery had not reached their mean baseline step count and MVPA five weeks after surgery. However, determining whether or not each individual patient has reached his or her own baseline step count, shows that only 61.5% (8/13) of the patients who underwent minor surgery and 25% (3/12) of the patients who underwent intermediate surgery, had reached their baseline step count or MVPA at the end of the study (five weeks after surgery). An explanation for this dissimilarity is that eight patients had surpassed their baseline levels five weeks after surgery, which increased the mean group levels. The outcomes of the accelerometer showed a fair agreement with the self-reported activity results recorded in the activity diary and a moderate agreement with patients’ report of having the feeling of being physically recovered.

There are few other studies which use the accelerometer in the postoperative care. In a study by Bisgaard et al. [18], 24 patients undergoing laparoscopic cholecystectomy used a wrist-worn mini-motion logger Actigraph accelerometer (Basic model, Ambulatory Monitoring, Inc, New York, NY) one week before surgery until one week after surgery [18]. Patients reached their baseline physical activity level two days after surgery. The difference in outcome might be explained by differences in study population. The pre-operative activity level in our study was lower than the pre-operative activity level in Bisgaards’ study (105.07 activity counts/minute vs around 200 activity counts/minute), which suggests that the study population in Bisgaards’ study consisted of a more active group. However, we have to be careful comparing the activity levels from both studies, since different measuring instruments are used. Secondly, our group of patients who underwent minor surgery, comprised three different types of laparoscopic surgery, of which three out of 13 underwent a laparoscopic cholecystectomy. The heterogeneity of our group might result in different outcomes. Lastly, the study group of Bisgaard et al. followed a relatively intense study program which might have resulted in a difference of motivation.

Another study carried out with an accelerometer in postoperative care by Wasowicz-Kemps et al. including an intervention group (*n* = 36) and a control group (*n* = 28) which both wore an accelerometer (PAM Model AM101, PAM B.V., Doorwerth, The Netherlands), performed measurements one week before a laparoscopic cholecystectomy until one week after surgery [19]. The control group received no feedback from their accelerometers as the display was turned off, while the intervention group did receive physical activity feedback from the display after surgery and also received personal advice regarding their physical activity. Activity scores were expressed in PAM scores based on accelerations in the vertical axis. In the control group 10/28 (36%) patients had reached their preoperative value one week after surgery compared to 18/36 (50%) patients in the intervention group. Reaching baseline was defined as a PAM score exceeding 90% of the mean preoperative value. In our study only 2/13 patients (15.4%) who underwent minor surgery reached their baseline step count one week after surgery and 5/13 (23.1%) their baseline MVPA. In our study the definition of reaching baseline was also set at 90% of the mean preoperative value. Again, the difference in outcome might be due to the heterogeneity of our minor surgery group and the fact that patients from the intervention group of Wasowicz-Kemps’ study received extra advice regarding their physical activities and could not see their own activity results.

Until now, our pilot study has been the first study in which postoperative physical activity levels are measured for a period longer than one week after surgery with an accelerometer, without any additional interventions, resulting in a clear and objective view of the recovery process. Only in obesity surgery, studies have been performed measuring activity levels after bariatric surgery nine months and 12 months after surgery respectively [28, 29]. However, these studies are focusing on measuring physical behavior changes instead of measuring recovery of physical activities after surgery. Another strength of our study is that we also measured self-reported activity results, using a self-developed activity diary. We have chosen to do so since pre-existing questionnaires focusing on daily activities were not suitable to match to the accelerometer results, because we were interested in a date of resumption after surgery and this is not what these kind of questionnaires focus on. We tried to develop a well-documented list, which has not yet been validated in this population, by using a functional ability list which earlier had been used to develop the convalescence recommendations of the type of surgical procedures we included in our study [24, 25]. However, since the agreement between the accelerometer results and the activity diary was fair in this study, no assumptions can be made regarding the fact that this was because we have not used a validated questionnaire to measure activities or because the correlation between self-report and objective measures was indeed low. This could be the case since the accelerometer only measures objective physical data and the self-report also contains subjective mental feelings regarding the recovery process. The same applies to the moderate agreement between patients’ self-report of being physically recovered and reaching baseline step count with the accelerometer. Future research in this field should therefore be performed using a validated physical activity instrument (such as the International Physical Activity Questionnaire [30]) alongside the accelerometer. Another limitation of our pilot study is the heterogeneity within the small study sample with regard to surgical procedures. Different surgical procedures might result in different patterns of recovery. However, the heterogeneity could also be regarded as an advantage, as using an accelerometer to objectively assess postoperative recovery levels might be applicable to a wide range of surgeries. We have tried to remedy this by dividing the surgical procedures into minor and intermediate surgery. Although the subdivision is partly based on current literature, the subdivision remains controversial. Therefore, future studies should try to include more participants per surgical procedure in order to improve comparison between and within the different patient groups. Another limitation is that the mean wear time per day differed significantly between the measuring weeks. Wear time during T1 was lower, which was due to the alternative definition that was used for this time point as many patients were bedridden and not always wearing the accelerometer in bed in this week. This may give an underestimation of physical activity in the first postoperative measuring week, especially in patients that might not have been bedridden and did not wear the accelerometer. However, wear time in the first week after surgery was considerably lower than in the other weeks mostly because participants did not wear the accelerometer in bed, and hence a correction for wear time would overestimate the physical activity levels in the first week considerably as non-wear time was mostly spent sedentary. Overall the protocol adherence was good, which suggests that the underestimation will have been minimal. Wear time during T2 and T3 was also lower than during T0, however in our opinion this difference was clinically not relevant and possibly still due to extra sleep time. Lastly, there was a relatively low inclusion percentage. The non-response analysis showed no significant differences in patient characteristics between included and non-included patients, but the included patients are likely to be a selection of the more motivated patients who are more willing to accept the additional burden of wearing an accelerometer.

This study has shown that the accelerometer can be used as an objective tool to measure recovery of physical activity levels after laparoscopic abdominal surgery (hysterectomy, adnexal surgery, cholecystectomy and hernia inguinal surgery). Step count and MVPA showed the same postoperative pattern which suggests that measuring postoperative step counts is an adequate measure for assessing postoperative activity level. Although this study showed that it is possible to measure postoperative recovery by an accelerometer, the clinical application remains controversial. First of all, because of the fact that using the accelerometer is relatively time consuming. This was supported by the low inclusion percentage in this study and the fact that although all patients completed the study, four patients could not fulfilling the strict wearing time criteria. A small wrist-worn device might have the advantage over the current hip-worn device, being easier to wear during the bed bound period just after surgery, resulting in better wear compliance. In addition the accelerometer which we used in this study costs approximately EUR 250 per device. Researchers can consider using a cheaper commercially available activity tracker which only measures steps in future research or clinical practice as opposed to our more expensive research based tool. Further, we think that the relatively low inclusion percentage was also because we could not offer the participants any benefit since they did not receive any feedback in the current study. However, when an intervention will be added, ie when patients can see their own activity results and get feedback, the added value might become obvious. The results of this study can help defining some standard values for giving feedback. In conclusion, an accelerometer can be useful in postoperative care to measure postoperative physical activity levels. Future research with a larger number of patients, also including open abdominal surgical procedures have to be performed to get more insight into the generalizability and clinical applicability of the results of this proof of concept study.

## Conclusions

This observational proof of concept study showed that:

1. The accelerometer showed a clear recovery curve over time and a clear difference in recovery curve according to level of invasiveness of the surgical procedure, which makes it a promising tool to assess post-operative recovery of daily physical activity levels.
2. The agreement between accelerometer results and the self-reported recovery times was fair in this study. More research is necessary to explore this.
3. The accelerometer was a feasible measure for objectively assessing post-operative recovery of daily physical activity levels, as it was well tolerated by the patients.

## Additional files

Additional file 1: Activity diary. The activity diary in which was completed by the patients. (DOCX 21 kb)

Additional file 2: Feasibility questionnaire. The questionnaire in which the feasibility of the accelerometer was evaluated. (DOCX 14 kb)

## Abbreviations

ASA: American society of anesthesiologists classification
BMI: Body mass index
MVPA: Moderate and vigorous intensity physical activity
SES: Social Economic Status. Scores are based on geographic location
