# Supplementary material for: Assessing pre- and postoperative activity levels with an accelerometer: a proof of concept study
Source: BMC Surg. 2017 May 12;17:56. doi: 10.1186/s12893-017-0223-0 (PMC5427573; doi:10.1186/s12893-017-0223-0)
Supplement: Supplementary file 1 — Activity diary. The activity diary in which was completed by the patients. (DOCX 21 kb) [file 12893_2017_223_MOESM1_ESM.docx]

**Activity diary** (this is a translation into English of the original Dutch version)

It is important for us to find out when you could resume specific activities after surgery. Therefore, we will ask you to use the table below during your recovery process. When you feel that you have returned your normal level regarding an activity, we ask you to fill in the date on which that happened, until you have completed all 20 items. When an activity is not applicable to tour situation, you may indicate this with an X.

| **Activities** | **Resumption date**  **(the date when you can perform the activity without restrictions)** |
| --- | --- |
| Sitting down for 2 hours |  |
| Sitting for the greater part of the day |  |
| Standing upright for 1 hour |  |
| Standing upright for the greater part of the day |  |
| Walking for the greater part of the day |  |
| Walking fast/running ^1)^ |  |
| Bending ^2)^ |  |
| Lifting: ± 15kg ^3)^ |  |
| Climbing stairs |  |
| Driving a car |  |
| Cycling |  |
| Household chores – light ^4)^ |  |
| Household chores – heavy ^5)^ |  |
| Shopping |  |
| Concentrate ^6)^ |  |
| Having the feeling of being recovered |  |
| Having the feeling of being fully mentally and physically recovered |  |
| Sports |  |

**You only have to fill in this table when you have a paid job:**

| Date of surgery | **… - … - …** |
| --- | --- |
| Date of the first day of resumption of work (also when this was only one hour) | **… - … - …** |
| Date of full resumption of work activities | **… - … - …** |

**For example:**

1. Running to catch a bus
2. Putting on socks and shoes, picking up something from the floor
3. A toddler, carrying a heavy grocery bag
4. Dishwashing, doing the laundry, sweeping the floor, vacuuming
5. Heavy carrying, performing chores, gardening
6. Reading, watching a documentary on television

We kindly ask you to return this list **5 weeks after surgery** by the attached return envelope (together with the accelerometer).
