# Supplementary material for: Assessing pre- and postoperative activity levels with an accelerometer: a proof of concept study
Source: BMC Surg. 2017 May 12;17:56. doi: 10.1186/s12893-017-0223-0 (PMC5427573; doi:10.1186/s12893-017-0223-0)
Supplement: Supplementary file 2 — Feasibility questionnaire. The questionnaire in which the feasibility of the accelerometer was evaluated. (DOCX 14 kb) [file 12893_2017_223_MOESM2_ESM.docx]

**Additional file 2 – Feasibility questionnaire** **(this is a translation into English of the original Dutch version)**

1. Was it clear for you how to use the accelerometer?
a Yes
b No

2. Did you find it a burden to wear the accelerometer?
a. Very
b. Slightly
c. Not really
d. Not at all

3. Were there occasions when you did not wear the accelerometer?
a. No
b. Yes one or two days
c. Yes more than two days
d. Yes a whole week

4. Reasons for not wearing the accelerometer
a. Forgot it
b. Too painful
c. Other: ……….
